# Art of Learning – An Art-Based Intervention Aimed at Improving Children’s Executive Functions

**DOI:** 10.3389/fpsyg.2019.01769

**Published:** 2019-07-31

**Authors:** Per Normann Andersen, Marita Eggen Klausen, Erik Winther Skogli

**Affiliations:** ^1^Faculty of Social and Health Sciences, Inland Norway University of Applied Sciences, Lillehammer, Norway; ^2^Faculty of Education, Inland Norway University of Applied Sciences, Lillehammer, Norway; ^3^Division of Mental Health Care, Child and Adolescent Psychiatric Clinic, Innlandet Hospital Trust, Lillehammer, Norway

**Keywords:** Art of Learning, behavioral self-regulation, BRIEF, executive function, executive function training, metacognition

## Abstract

Executive functions (EFs) can be conceptualized as a mean of behavioral self-regulation, and difficulties with EFs may adversely affect school success, social function, and cognitive and psychological development. Research about EFs and how they are affected by various educational and psychosocial factors is sparse. EFs are of great importance to understand how children can handle the challenges that they meet at various stages of development. There has been an increased focus on programs aimed at improving EFs, either as a primary outcome, or as a supplemental result of a specific activity. In this randomized controlled study, 66 children (31 girls, mean age 7:1 years) were given an arts and culture rich intervention (Art of Learning) aimed at improving EFs. EFs were assessed with the Behavior Rating Inventory of Executive Functioning-teacher version (BRIEF-teacher form) before, immediately after, and 6 months after intervention. Outcome in the intervention group was compared to children from two schools serving as controls (*n* = 37, 18 girls, mean age 7:3 years). In addition, teachers from intervention schools were also interviewed both individually and in focus groups. The results reveal that both groups improved their EFs, as measured with BRIEF, over time on the global executive composite (GEC) score, the metacognition index, and on behavioral regulation index (BRI). However, the intervention group displayed a significantly greater improvement than the control group on GEC and BRI. The teacher interviews reveal positive effects for the children when it comes to several aspects: collaboration, conflict management, inclusion, vocabulary, and confidence. These factors are regarded as important for EFs development and academic outcome. The results support the notion of best training transfer effects for tasks addressing global executive functioning and specifically behavioral regulation skills (BRI).

## Introduction

Executive functions (EFs) can be conceptualized as a mean of behavioral self-regulation, crucial for children’s social function, and cognitive and psychological development (Alloway, 2009). EFs seem to be situated in neural networks including prefrontal cortex, striatum, and the basal ganglia (Middleton and Strick, 2001), showing considerable development throughout childhood, reaching adult-like levels in middle adolescence (Best and Miller, 2010). Thus, difficulties with EFs have shown to adversely affect school success, social function, and cognitive and psychological development (Diamond, 2013; Zelazo et al., 2016; Pellicano et al., 2017; Morgan et al., 2018). There are no generally agreed definition of EFs. One reason for this might be different research approaches to the construct, either through studies of functional outcome of frontal lobes lesions/damage, or examining different cognitive functions thought to regulate goal-directed behaviors and studies investigating development of cognitive control strategies and self-regulation (Cirino et al., 2018). However, a definition commonly used is provided by Miyake and Friedman (2012) and refer to EFs as “*a set of general-purpose control processes that regulate one’s thoughts and behaviors*”. Another common conceptualization refer to EFs as “*the attention-regulation skills that make it possible to sustain attention, keep goals and information in mind, refrain from responding immediately, resist distraction, tolerate frustration, consider the consequences of different behaviors, reflect on past experiences, and plan for the future*” (Zelazo et al., 2016).

There is some ambiguity as to whether EFs can be judged as a unitary construct or as a set of independent components. Miyake et al. (2000) has gained a lot of support in their effort to bridge the different constructs into a unity/diversity hypothesis of EFs. In this view, EFs are both related and separate cognitive functions. The unity/diversity hypothesis of EFs finds evidence for both distinct and common loadings of inhibition, working memory (WM), and cognitive flexibility in EFs (Miyake et al., 2000; Best and Miller, 2010; Diamond, 2013). These functions are regarded as foundations for other higher-order cognitive skills, such as reasoning, problem solving, and planning (Diamond, 2013).

Inhibition comprises cognitive functions such as self-control, selective/focused attention, and cognitive inhibition. Inhibitory control improves rapidly in early childhood, followed by a less dramatic change through adolescence (Best and Miller, 2010). Poor inhibitory control is associated with reduced quality of life, and relatively small improvements may have huge gains (Moffitt et al., 2011).

The most commonly used definition of WM defines the construct as the active maintenance and manipulation of information within a limited time span (Baddeley, 2003). WM have been shown to be crucial for children’s learning capacity, and academic achievement in school (Alloway et al., 2005; Alloway, 2009). As the development of WM is closely related to the maturation of inhibitory control, the developmental trajectory of WM is often difficult to disentangle from inhibition (Best and Miller, 2010). That said, converging evidence indicate a more protracted period of development for WM, showing improvement at least through adolescence (Best and Miller, 2010).

Cognitive flexibility refers to the ability to adapt to changing situations requiring different thoughts and behaviors (Hill, 2004). The failure in generating novel solutions and to use appropriate levels of representations in mental processing may hinder creative responses in situations with open-end outcomes (Ridley, 1994). Cognitive flexibility is important for the behavior required in daily social activities (Memari et al., 2013). Cognitive flexibility presupposes inhibitory control and WM showing a protracted period of development through adolescence (Best and Miller, 2010).

The notion of EFs as interrelated, but at same time distinct components are also supported in a recent study by Cirino et al. (2018). When assessing EFs in 846 children from 8 to 12 years they found a common factor and five separate factors. Two components were WM related; span/manipulation with planning and updating. The other three were generative fluency, self-regulated learning (SRL), and metacognition (MCOG). The EFs trident of WM, inhibition, and cognitive flexibility from the unity/diversity model of EFs by Miyake et al. (2000) was not supported, which is also the case in other studies evaluating model fit in children (Huizinga et al., 2006; Espy et al., 2011; Lee et al., 2013; but see Miyake and Friedman, 2012). The two WM components found by Cirino et al. (2018) fit well into the constructs of WM given above. SRL can be described within the concept of EFs given by Zelazo et al. (2016) comprising planning, reasoning, and problem-solving abilities. MCOG refers to the ability to monitor, manipulate, and regulate other cognitive processes (Cirino et al., 2018). The ability to monitor and regulate cognitive processes has been a central feature of the EFs models given by Barkley (1997). EFs can also be conceptualized as a mean of behavioral self-regulation where inhibition in particular has been associated with childhood aggression (Barkley, 1997; Poland et al., 2016). A metacognition index (MI) is incorporated as a main scale in the Behavior Rating Inventory of Executive Functioning (BRIEF), contrasting the behavioral regulating abilities in the Behavioral Regulation Index (BRI) (Gioia et al., 2000a). Behavior regulation have in previous studies been associated with social function (Kenworthy et al., 2009), while metacognitive skills may be of greater importance for school performance (Carretti et al., 2014). Although describing the concept of EFs with different operationalization’s, the above-mentioned descriptions seem to entail some of the same cognitive mechanisms. The overarching notion of EFs as a cognitive process regulating thoughts, behavior, and emotions important for everyday functioning seems to be unanimous. And as early EFs functioning predicts later EFs functioning (Moffitt et al., 2011), interventions aimed at improving EFs are important.

The link between EFs and creativity is somewhat debated. Radel et al. (2015) found that less inhibitory control was associated with more fluent generation of ideas, one central aspect of creativity. On the other hand, being able to cognitively inhibit unrelated ideas is found to improve ideational fluency and flexibility (Benedek et al., 2012). The modulation of defocused attention together with controlled processing/selective focused attention can be regarded as processes needed for cognitive flexibility which is associated with creativity (Zabelina and Robinson, 2010).

Several approaches, both direct and indirect interventions aim to increase EFs in children. These approaches span from games, digital games, art programs, social pretend play, mindfulness, physical exercise, martial arts to parent training, and specific educational practices (Diamond, 2012; Hsu et al., 2014; Zelazo et al., 2016). Evidence for effects are mixed and are usually measured with neuropsychological measures with relatively low correlation to everyday EFs as it unfolds in the classroom (Toplak et al., 2013). Several pedagogical practices have shown evidence for improving EFs in children; however, the evidence for these are also mixed (for review see Jacob and Parkinson, 2015; Zelazo et al., 2016). Further, the unity/diversity hypothesis by Miyake et al. (2000) also raises the question whether different intervention programs will show best effect on behavioral self-regulation, metacognitive skills, or result in more global EFs improvements.

The best evaluated of these programs, “Tools of the Mind,” “Head Start REDI,” and the “Chicago School Readiness Program (CRSP)” are designed for kindergarten. Malleability of EFs is thought to be best in pre-school years (Diamond, 2014). The “Promoting Alternative Thinking Skills” program (PATHS; Greenberg et al., 1995) is to our knowing the only program designed for elementary school. PATHS is designed to promote emotional and social functioning, and to reduce behavior problems. This focus is thought to improve EFs as well (Riggs et al., 2006). Bierman et al. (2008a) reported improved emotion-regulation and social problem solving in a randomized controlled trial (RCT) of 356 pre-kindergarten children enrolled in PATHS curricula (Bierman et al., 2008a). Small to moderate effects of this RCT was further reported for improved examiner ratings of the children’s attention, and performance on a neuropsychological test assessing EFs (i.e., the dimensional Card Sort Task) (Bierman et al., 2008b). In older age groups, however, effects of PATHS school curricula are reported to be smaller (*d* = 0.1–0.2) than for pre-kindergarten studies (Morris et al., 2014).

Art of Learning (AoL) is a program that combines teacher professional development with a children’s learning program over a period of 12 weeks (Creativity Culture and Education, 2018). The AoL program hypothesizes that an arts rich, creative learning program, delivered intensively in schools over several weeks can have a positive impact on the development of EFs and attainment in children (Creativity Culture and Education, 2018). AoL aims to improve teachers understanding of creative skills and EFs. Furthermore, to help teachers gain more confidence in using arts-based approaches and learning to improve attainment across the curriculum. AoL seeks to improve children’s understanding of their own creativity and help them develop their EFs. The activities in the program focus on each of the following art forms: music, theatre/drama, dance, literature/poetry, visual arts, and photography/digital art. AoL is largely based upon the review of the existent literature by Diamond (2014), giving evidence that EFs interventions using arts and physical activities are most promising. AoL has not yet been evaluated.

Executive functions are usually assessed through laboratory-based neuropsychological testing, measuring optimal performance at a given time and with very limited distracting stimuli. Hence, laboratory-based testing may not adequately represent how children are able to utilize their EFs in the complexity of more naturalistic settings, and questions have been raised about the ecological validity and generalizability of neuropsychological test results (McCue and Pramuka, 1998). Furthermore, assessing EFs using neuropsychological test batteries is also time-consuming and costly. The BRIEF, which is used in this study tries to accommodate this critique aiming to measure EFs abilities needed for everyday adaptive behavior and functioning through teacher completion of the BRIEF rating scale (Gioia et al., 2000a). This together with interviews with participating teachers trying to capture both near and far transfer effects of the intervention.

The first aim of the current study was to examine whether an arts rich intervention constructed to improve children’s EFs would yield any effect on a measure on everyday EFs as reported by children’s teachers, and as reported in interviews. Based on current knowledge we hypothesized that the intervention group would have a greater improvement overall in everyday EFs than the children in the control group. Our second aim was to delineate whether this intervention program, delivered intensively in schools, will have a differential impact on behavioral self-regulation and MCOG. According to findings reported by Carretti et al. (2014) we hypothesized that the group receiving intervention will show a greater improvement in MCOG than the control group. We did not expect to find greater improvement in behavioral self-regulation in the group receiving intervention compared to the control group.

## Materials and Methods

### Participants

A total of 103 children (49 girls) between 6.1 and 9.3 years (Table 1) were recruited from five different public schools in the rural area of Gudbrandsdalen in Norway. Children from three schools (grades 1–2) received the 12-week long AoL intervention and children from the last two schools (grades 1–3, see Table 2) served as a control group. Children in the control group worked with their curricula in a traditional manner and received no specific intervention during the trial period. All schools had volunteered to participate in the study. The schools were randomly selected to either intervention or control conditions. At baseline (T1), EFs for all children were assessed by their teachers with the BRIEF-teacher form (Gioia et al., 2000a). The same teachers assessing children’s EFs at T1 also reassessed them post-intervention (T2) and after 6 months (T3). Demographic characteristics are presented in Tables 1, 2.

**TABLE 1:** Demographic characteristics.

Variable	Intervention (*n* = 66)	Control (*n* = 37)	**Group comparisons**	
			χ^2^**/*F***	p
Sex (male/female)	35/31	19/18	0.27	NS
Age (months)	85.2 (6.1)	88.5 (9.8)	(1,101) 2.12	NS
BRIEF – GEC T1	92.1 (20.9)	87.9 (20.5)	(1,101) 0.96	NS

*BRIEF – GEC T1, Behavior Rating Inventory of Executive Function – Global Executive Composite Time 1; NS, not significant.*

**TABLE 2:** Number of participants in different grades and numbers of interviews conducted in parenthesis.

		**Group**		**Total**
		**Intervention**	**Control**	
Grade	1,00	30 (3)	17	47
	2,00	36 (3)	13	49
	3,00	0	7	7
Total		66	37	103

Focus group interviews with teachers were conducted at the intervention schools, as well as individual interviews with one teacher at each of these schools. Three focus group interviews and three individual interviews in total. Strategic committees were made by discussing with the principals of two of the schools, which teacher had been active in the project all the way, and who had qualifications to be able to provide good information in the interview. At the third school, only one of the teachers had time to join the interview. Therefore, an accessibility selection was made there. That is to say – the sample was strategically based on the fact that the participant represented properties that were relevant to the problem, and the method for selecting this teacher was based on the teacher being available. All the teachers met up at the agreed time to a 1.5 h per group interview and 1 h per individual interview.

### Art of Learning

Art of Learning is an arts rich, creative learning program delivered intensively in schools and aims to have an impact on the development of creative skills, EFs, and attainment in children (Creativity Culture and Education, 2018). AoL is a practice-based program where artists work in partnership with teachers to support planning and implementation of lessons. The program has a duration of 12 weeks and comprises pre-designed creative learning practices from six different artforms (music, theatre/drama, dance, literature/poetry, visual arts, photography/digital) delivered 1 h (60 min) a day 3 days a week (see Supplementary Data Sheets 1–6 for examples). Each activity is specially designed to address either one or more of the EFs; inhibition, WM, or mental flexibility. The sessions consisted of a selection of 36 predetermined art activities and were translated and adapted to the Norwegian context. They involved a large upheaval of everyday life for the intervention group, while for the control group it meant having teaching as normal. The artists came to the intervention schools and conducted the predetermined arts activities with the children in collaboration with the teachers. The artist and the teacher themselves, designs and deliver a 1-h activity (60 min) each week based on the experiences they gain from the program. The artists’ work in one class over a period of 6 weeks and then another artist follows the class for the remaining 6 weeks. The children received a total of 240 min, or 4 h of arts activities each week, through the 12 weeks. The sessions were structured based on the children engaging with activities from the different art forms. Each art form (music, theatre/drama, dance, literature/poetry, visual arts, photography/digital) was devoted to 6 sessions, or 2 weeks.

The sessions were built up according to a fixed structure: warm-up, main activity, and reflection. Each session schedule provided instructions on time usage, materials needed, room setup, guidance on how to conduct the activity, and which EFs the session aimed to train (Table 3). It was up to the artists and the teachers to adapt the sessions to the group of children. The artists recorded all changes from the original plans after each session and have since been filed in the project database.

**TABLE 3:** Overview over Art of Learning exercises and aimed executive functions.

**Artform**	**Session**	**Warm up**	**IC**	**WM**	**CF**	**Main activity**	**IC**	**WM**	**CF**	**Reflection**	**IC**	**WM**	**CF**
Dance	Week 1 Session 1	Dance warm up	✓			Name dance		✓	✓	Questions			✓
	Week 1 Session 2	Dance warm up 2	✓	✓		Movement symmetry	✓	✓	✓	Mindful breathing	✓		
	Week1 Session 3	Dance warm up 3	✓	✓		The match moves		✓	✓	Questions			✓
	Week 2 Session 1	Brain warm-up and SG	✓		✓	Welcome to the circus	✓		✓	Open circle			✓
	Week 2 Session 2	Alive, once alive, never..			✓	Welcome to the rainforest	✓		✓	Postcard partners		✓	
	Week 2 Session 3	Stop go weather game	✓		✓	It’s raining, it’s pouring			✓	Think, pair and share	✓		✓
Literature	Week 1 Session 1	Shoulders	✓	✓		This is a haiku		✓	✓	Scale game		✓	
	Week 1 Session 2	This is a…			✓	Be very afraid			✓	Step in			✓
	Week 1 Session 3	Poetry clap	✓	✓		Maths poetry		✓	✓	One word		✓	✓
	Week 2 Session 1	Group story with cards		✓	✓	Emotional fiction			✓	Walking emotions			✓
	Week 2 Session 2	Group story with cards 2	✓	✓	✓	Fifty-word story	✓		✓	Walking reflection	✓		
	Week 2 Session 3	Group story with cards 3	✓	✓	✓	Fifty-word story 2	✓		✓	Moving reflection	✓		
Music	Week 1 Session 1	Four beats	✓			Beat games	✓		✓	Questions			✓
	Week 1 Session 2	Don’t clap this one back	✓	✓		Louisiana mud slap	✓	✓		High and low reflection			✓
	Week 1 Session 3	Ta ta kidi	✓		✓	Rhythm of my body	✓	✓		Feeling through my body			✓
	Week 2 Session 1	Plasticine person	✓	✓	✓	Beatboxing	✓	✓		Sound reflection			✓
	Week 2 Session 2	Rhyming stamp	✓		✓	Rapping and rhyming			✓	Reflecting on our work			✓
	Week 2 Session 3	The opposite game	✓	✓		Putting on a show		✓	✓	Dartboard reflection			✓
Theatre	Week 1 Session 1	Stop, go, gettingtoknow		✓		Daily routine disco	✓		✓	Questions	✓	✓	✓
	Week 1 Session 2	Yes, let’s			✓	The bag part 1		✓	✓	Freeze frame		✓	✓
	Week 1 Session 3	1,2,3	✓	✓	✓	The bag part 2		✓	✓	Scale game	✓	✓	✓
	Week 2 Session 1	Stop go			✓	Mask monologs		✓	✓	Mask monol. on paper	✓	✓	
	Week 2 Session 2	Fast and freeze	✓		✓	What it’s like to be…			✓	Questions		✓	
	Week 2 Session 3	Speed graffiti		✓	✓	What it’s like to be… 2			✓	Open-minded reflection			✓
Visual arts	Week 1 Session 1	Big draw	✓			Back to back	✓		✓	Eyes closed	✓	✓	
	Week 1 Session 2	Memory draw		✓		Simon says – collage create	✓	✓	✓	Facial feedback		✓	
	Week 1 Session 3	Question square			✓	Frames of reference	✓		✓	I liked	✓	✓	
	Week 2 Session 1	Count to 20	✓			Picture in my mind	✓	✓		Recalled reflections		✓	
	Week 2 Session 2	Hand squeeze	✓			Drawing through my senses	✓	✓	✓	Post-it feedback			✓
	Week 2 Session 3	Changing spaces	✓	✓		Portrait of change			✓	Scale game			
Digital	Week 1 Session 1	Me-pose	✓		✓	Picture story	✓		✓	Paper-ball free-writing	✓		✓
	Week 1 Session 2	Speed graffiti			✓	Picture an emotion			✓	Emotional questions in pairs	✓		
	Week 1 Session 3	Group story		✓	✓	Sound story of origins		✓	✓	Radio interview			✓
	Week 2 Session 1	Silent walk	✓			School advert 1			✓	Yes/no questions			✓
	Week 2 Session 2	Bouncy warm-up			✓	School advert 2	✓		✓	Bouncy reflection	✓		✓
	Week 2 Session 3	Nod, shrug and shake	✓			School advert 3	✓		✓	Bottle reflection			✓

*IC, inhibitory control; WM, working memory; CF, cognitive flexibility.*

Principals and teachers from the intervention schools, as well as the artists, were trained ahead of the intervention to ensure that they understood and could conduct the practical aspects and the content by being part of the AoL, introducing them to the aims and explaining the different elements of the program (see Supplementary Appendix 1). They were also given a comprehensive lecture of EFs and its relation to learning and creativity. Teachers from control schools were not given this information or training, this to ensure they did not alter their pedagogical practices accordingly.

Artists who were to carry out the activities were recruited based on experience from previous, similar activities in schools. They were placed at the various intervention schools on the basis of a desire for continuity. It was stressed that the artists should become acquainted with the children and the teachers and vice versa, based on which arts the artists worked with. Planning time for the artist and teachers at least once a week was provided. How the days were organized, and when time was allocated for planning varied from school to school. The schools were given all the material they needed before the intervention period, except for material they had easy access to at each school. Otherwise, the artists were responsible for ensuring that all material was ready before each session, and for preparing it. During the intervention, the Project Leader visited all the schools to ensure program fidelity.

### Measures

The BRIEF rating scale (5–18 years) assesses everyday executive functioning and provides information about cognitive, emotional, and behavioral regulatory processes (Gioia et al., 2000b). BRIEF-teacher form is completed by the child’s teacher and contains 86 items measuring different empirically derived aspects of EFs behaviors. These are Inhibit, Shift, Emotional Control, WM, Initiate, Plan/Organize, Organization of Materials, and Monitor. These eight clinical scales form two broad classifications of executive functioning; Behavioral Regulation (BRI) and Metacognition (MI), as well as an overall Global Executive Composite (GEC) score (Gioia et al., 2000a). The current study used the Norwegian version of the teacher form. The teacher form has shown high internal consistency with a Cronbach’s α ranging from 0.80 to 0.98, and with test–retest reliability correlations for BRI = 0.92, for MI = 0.91, and for GEC = 0.91. Further, correlational analysis provide evidence for convergent and divergent validity through comparisons with other established scales for behavior (Gioia et al., 2000a). Studies have reported discrepancies comparing European children with the American norm sample in favor of European children scoring better than American norms (Fallmyr and Egeland, 2011; Huizinga and Smidts, 2011; Hovik et al., 2014). Of interest in the current study are the broad measures BRI, MI, and the overall GEC. Raw scores are used in the analyses. Lower raw scores on the BRIEF indicate better EFs. The teachers completing the BRIEF were the same teachers who led the intervention in the classroom together with the artists.

Information from BRIEF-teacher form was supplemented with a partially structured interview, in which the questions and topics are pre-arranged, but with the opportunity and openness for the informants’ experiences as well as room for follow-up questions along the way.

### Analysis

SPSS version 24 was used for statistical analysis. Significant results are reported at the *p ≤* 0.05 level. Demographic characteristics are investigated using chi-square test for independence (gender) and independent samples *t*-test (age). Mixed between-within subjects’ ANOVA (mixed ANOVA) were used to investigate possible interaction effects in EFs development across groups (intervention vs. controls, girls vs. boys). Significant interaction effects were followed up with repeated-measures ANOVA for each group. Indications of violations of the assumption of sphericity will be reported together with Greenhouse–Geisser corrected tests of within-subjects’ effects. Significant interaction effects from mixed ANOVAs were also followed up with paired samples *t*-tests to investigate differences within groups between T1−T2 and T2−T3.

The semi-structured interview had eight different topics: aims, the sessions, executive functioning, academic functioning, social functioning, role of the teacher, methods, and improvements. Six teacher interviews with three 1st and three 2nd grade teachers from three schools with a duration of approximately 60–90 min were conducted. All the interviews were recorded using a telephone recorder before being transferred to a computer with anonymous titles. After all the interviews were completed, they were structured for analysis by transcription. All participants became anonymous in the enrolment. The computer program QDA Miner Lite was used for coding and categorization. In this process, several meaningful categories were extracted, and a selection of these was included in this report. The categories are related to statements made by the informants about the phenomena they had experienced along the way and after the intervention. The material was read and reviewed several times.

This study was carried out in accordance with the recommendations of the Norwegian Centre for Research Data with written informed consent from parents of all subjects. All parents gave written informed consent in accordance with the Declaration of Helsinki. The protocol was approved by the Norwegian Centre for Research Data.

## Results

### Behavior Rating Inventory of Executive Function (BRIEF)

**FIGURE 1:**
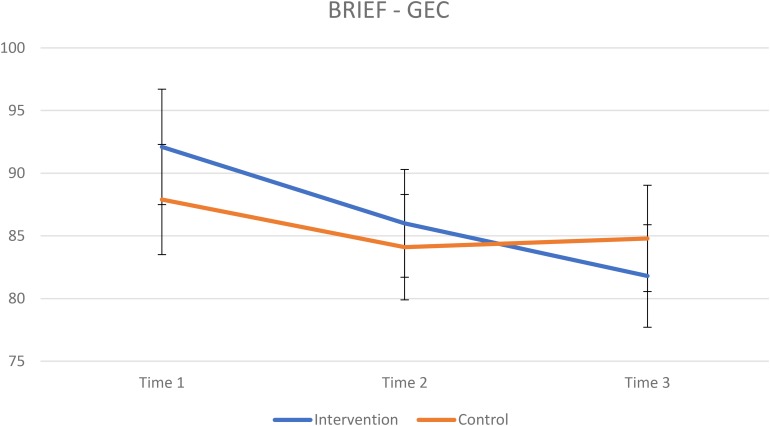
Raw scores, lower scores indicating better results. Vertical bars denote 95% confidence intervals.

**FIGURE 2:**
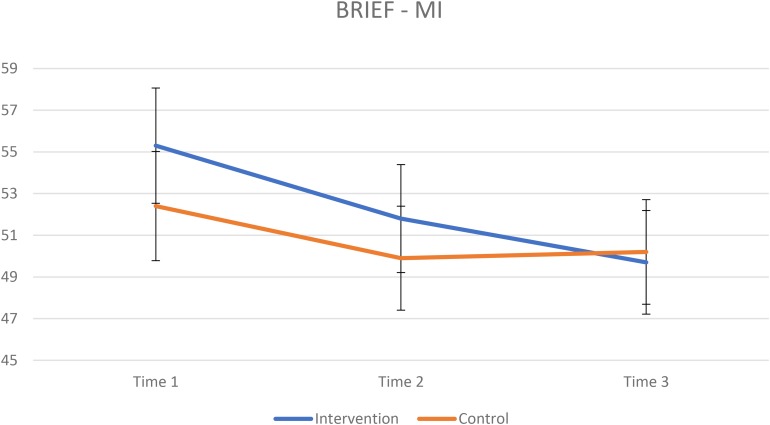
Raw scores, lower scores indicating better results. Vertical bars denote 95% confidence intervals.

**FIGURE 3:**
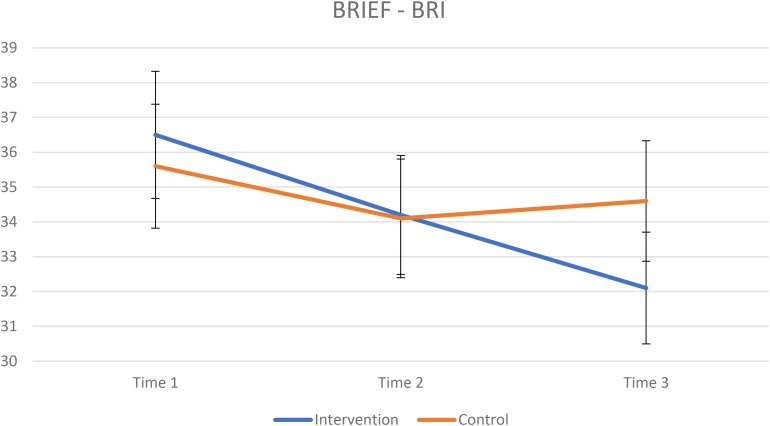
Raw scores, lower scores indicating better results. Vertical bars denote 95% confidence intervals.

**FIGURE 4:**
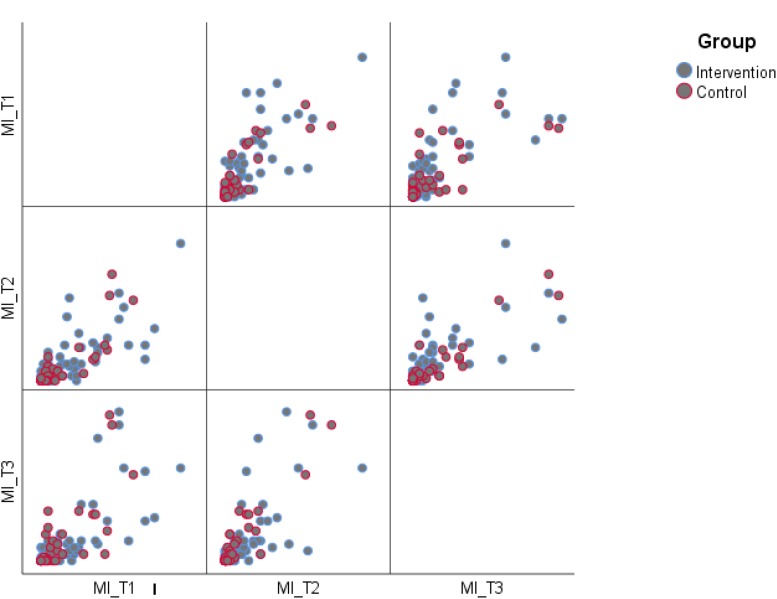
Scatterplot matrix metacognition index time 1, 2, and 3.

**FIGURE 5:**
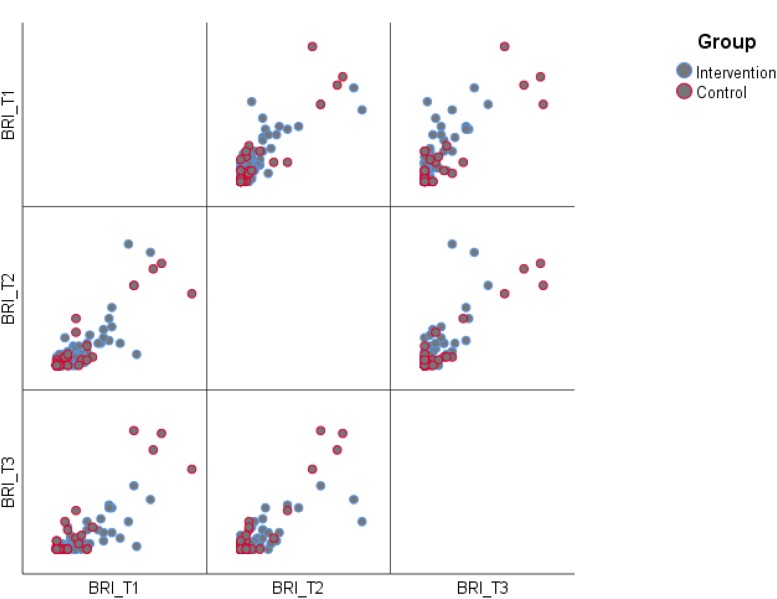
Scatterplot matrix behavioral regulation index time 1, 2, and 3.

The results from the BRIEF for the intervention group and control group are presented in Table 4. The mixed ANOVA for GEC revealed a significant interaction effect of group × time [*F*(2,202) = 4.4, *p* = 0.014, $\eta_{p}^{2}$ = 0.042] indicating greater improvement on results in favor of the intervention group (Figure 1). A repeated measure ANOVA for each group revealed that both had improved scores on GEC over time, intervention group: *F*(2,130) = 19.2, *p* < 0.001, $\eta_{p}^{2}$ = 0.228. For the control group Mauchly’s test indicated a violation of the assumption of sphericity, χ^2^(2) = 16.3, *p <* 0.001, results from Greenhouse–Geisser (ε = 0.73)-corrected tests: *F*(1.49,52.5) = 4.05, *p* = 0.035, $\eta_{p}^{2}$ = 0.101. A paired-samples *t*-test for the intervention group revealed a significant improvement from T1 to T2 [*t*(65) = 3.58, *p* = 0.001, *d* = 0.30] and from T2 to T3 [*t*(65) = 2.56, *p* = 0.013, *d* = 0.26]. A paired-samples *t*-test for the control group revealed a significant improvement from T1 to T2 [*t*(36) = 2.81, *p* = 0.008, *d* = 0.20] but not from T2 to T3 [*t*(36) = −0.7542, *p* = 0.456, *d* = −0.04]. There was no significant interaction effect of group × time on the mixed ANOVA for MI (Figures 2, 4). A significant effect of time was found for MI [*F*(2,202) = 11.9, *p* < 0.001, $\eta_{p}^{2}$ = 0.105]. The mixed ANOVA for BRI showed a significant interaction effect of group × time, *F*(2,202) = 5.3, *p* = 0.006, $\eta_{p}^{2}$ = 0.050 (Figures 3, 5). A repeated measures ANOVA shows a significant effect of time for BRI in the intervention group *F*(2,130) = 20.3, *p* < 0.001, $\eta_{p}^{2}$ = 0.237, but not for the control group *F*(2,72) = 1.85, *p* = 0.164, $\eta_{p}^{2}$ = 0.049. A paired-samples *t*-test for the intervention group revealed a significant improvement from T1 to T2 [*t*(65) = 3.42, *p* = 0.001, *d* = 0.27] and from T2 to T3 [*t*(65) = 2.96, *p* = 0.004, *d* = 0.30]. A paired-samples *t*-test for the control group revealed no significant improvement from T1 to T2 [*t*(36) = 1.81, *p* = 0.077, *d* = 0.14] nor from T2 to T3 [*t*(36) = −0.862, *p* = 0.378, *d* = −0.04].

**TABLE 4:** Results on BRIEF-teacher form (raw scores) at T1, T2, and T3: means and standard deviations within the intervention and control groups, and results from mixed model ANOVA.

**Variable**	**Intervention (*n =* 66)**			**Control (*n =* 37)**			**Group**		**Time**		**Time × group**		
	**T1**	**T2**	**T3**	**T1**	**T2**	**T3**	*F*	*p*	*F*	*p*	*F*	*p*	$\eta_{p}^{2}$
GEC	92.1 (20.9)	86.0 (18.6)	81.8 (13.7)	87.9 (20.5)	84.1 (19.6)	84.8 (21.7)	(1,101) 0.082	NS	15.8	>0.001	4.37	0.014	0.042
MI	55.3 (13.7)	51.8 (11.1)	49.7 (10.2)	52.4 (10.8)	49.9 (10.7)	50.2 (10.7)	(1,101) 0.504	NS	11.9	>0.001	2.24	NS	0.022
BRI	36.5 (8.31)	34.2 (8.61)	32.1 (5.00)	35.6 (11.8)	34.1 (10.1)	34.6 (11.8)	(1,101) 0.083	NS	12.6	>0.001	5.30	0.006	0.050

*BRIEF, Behavior Rating Inventory of Executive Function; GEC, global executive composite; MI, metacognition index; BRI, behavioral rating index; NS, not significant.*

We found no significant interaction effects between time, group, and gender on GEC, MI, or BRI [GEC, *F*(2,198) = 0.28, *p* = 0.755, $\eta_{p}^{2}$ = 0.003; MI, *F*(2,198) = 0.01, *p* = 0.988, $\eta_{p}^{2}$ = 0.000; BRI, *F*(2,198) = 1.54, *p* = 0.216, $\eta_{p}^{2}$ = 0.015]. Neither did we find any interaction effects between time and gender.

### Semi-Structural Interviews With Teachers

The coding and categorization process extracted the following categories relevant for the aims of this article.

#### Collaboration

The teachers from all three schools described that the project gave the children new tools to succeed in cooperation with others. The students had to discuss solutions, give and take, individualists had to open to others’ views and ideas. The teachers believed that intervening with classroom dynamics provided a better school environment.

#### Conflict Management

The teachers all report that the children’s abilities to resolve conflicts improved because of the intervention. At one school, the teachers report that both children and teachers have a new approach in conflicts, as the children have learned new concepts and tools to resolve conflicts, and the teachers have become better at challenging the children to reflect upon difficult situations. In another school, the teachers report improved generosity in the group, the children accept each other in a new way, and no one is laughing at each other.

#### Inclusion

In the interviews, the teachers emphasize the effect the project has had for inclusion. Some describes how the children, through collaborating with several, not just the best friends, create a better school environment. They see that children who were previously left out are included – that everyone is included in a different way than before. The established groups were dissolved, and the children expanded their circle of friends.

#### Vocabulary

The teachers all report that the children have expanded their vocabulary. They describe, respectively, how the children have acquired a richer language, learned new concepts, and have become more reflective. One teacher describes how the children gained a larger conceptual apparatus and thus were able to verbalize how they experienced the sessions. That children who initially just didn’t want to be involved at the end were able to verbalize their own internal conflicts and how they could solve it. Furthermore, teachers describe how children’s ability to take conversational turns also had improved.

#### Confidence

All schools describe that the children have become more confident in expressing their own opinions, and in taking responsibility for group achievements. Teachers from all schools believe the intervention has given the children mastery and a sense of increased self-confidence.

## Discussion

The results reveal that both groups improve their EFs, as measured with BRIEF, over time on the GEC score, the MI and on BRI. However, the group receiving the intervention had a significantly greater improvement than the control group on GEC and BRI. The teacher interviews reveal several effects of the project. They report positive effects for the children when it comes to; collaboration, conflict management, inclusion, vocabulary, and confidence. The results revealed no gender differences regarding development of EFs throughout the study period.

### Global Executive Composite

The first aim of the current study was to examine whether an arts rich intervention constructed to improve children’s EFs would yield any effect on a measure on everyday EFs, as reported by children’s teachers. Based on current knowledge we hypothesized that the intervention group would have a greater improvement overall in everyday EFs than the children in the control group. As expected, the intervention group displayed greater improvement overall with more than twice as large effect sizes, on a measure on everyday executive function (GEC) as reported by children’s teachers, compared to the control group.

These results indicating a global effect of AoL are consistent with previous research showing a significant transfer effect of school curricula aiming to enhance EFs (Diamond and Lee, 2011). These findings are also corroborated by the reports from the teachers involved in the intervention and their reflections upon the effects it had on the participants when it comes to social competence, verbal abilities, and self-assurance. One potential explanation for this wide transfer effect of school curricula programs such as AoL may be the emphasis on dynamic EFs training in all activities, across different situations that may stay in contrast to more specialized EFs programs showing less generalized effect (Lillard and Else-Quest, 2006; Riggs et al., 2006; Diamond, 2007). As can be seen from Figure 1, GEC improvement in the intervention group and the control group divert from each other from the timepoint that the intervention was discontinued and until follow-up after 6 months. However, it must be noted that this global effect on improved EFs in the intervention group compared to the control group may primarily be driven by improved BRI in the intervention group. As a main difference between groups across time was found for the BRI, improved BRI scores in the intervention group attributes to most of the overall GEC score based on both MI and BRI. There is a possibility that the teachers involved in the intervention gradually altered their pedagogical practices to be more in accordance with AoL, so the prolonged effect may be directly related to the intervention. Such learning gains for teachers have also been reported from previous evaluations of other creative, arts and culture rich school curricula (Thomson et al., 2015).

### Behavioral Self-Regulation

Our second aim was to delineate whether this intervention program, delivered intensively in schools, will have a differential impact on behavioral self-regulation and MCOG. According to findings reported by Carretti et al. (2014) we hypothesized that the group receiving intervention will show a significantly greater improvement in MCOG than the control group. We did not expect to find significantly greater improvement in behavioral self-regulation in the group receiving intervention compared to the control group. Contrary to our expectations, we did not find support for greater improvement in MCOG for the intervention group, compared to the control group. Surprisingly, the intervention group displayed a greater improvement in behavioral regulation with four times as large effect sizes compared to the control group. Thus, findings from the current study did not support our hypothesis that an arts rich intervention, constructed to improve children’s EFs would yield a particular effect on EFs sub-functions shown to be crucial for school performance (Carretti et al., 2014). Where the MI reflect the child’s ability to get engaged in planful and organized problem-solving, as well as, updating and shifting of information needed, the BRI to a higher extent comprises subscales reflecting the child’s ability to initiate, inhibit, and modulate behavior, emotions, and activities (Gioia et al., 2000b).

Interestingly, results from our study showing improved behavioral regulation are consistent with findings by Bierman et al. (2008a) reporting better emotion-regulation and social problem-solving skills in pre-kindergarten children after being enrolled in PATHS curricula. Along the same lines, the reduction in problem behavior (*d* = 0.53−0.89) was the main finding in one “CRSP” – RCT of 609 preschool children, showing more moderate improvements in academic skills (*d* = 0.20−0.63) (Raver et al., 2009, 2011).

A similar main effect on improved behavioral outcome is evident in our study. When inspecting paired-samples *t*-tests for the intervention group, the global effect of AoL from T1 to T2 (GEC: *d* = 0.30) and from T2 to T3 (GEC: *d* = 0.26) is mainly driven by improved BRI (T1−T2: *d* = 0.27; T2−T3: *d* = 0.30). The effect sizes of *t*-tests from T1 to T2 and T2 to T3 are also small, below, or in line with comparable studies mentioned above. However, the effect sizes from the mixed measures ANOVA’s are larger ($\eta_{p}^{2}$ = 0.237) and indicate that these children continue to improve their EFs more than controls from T1 to T3, although improvements are small to moderate. Albeit the findings from the RCTs by Bierman et al. (2008a), and Raver et al. (2009, 2011) are based on preschool children from low-income families, our findings corroborate previous results elucidating the centrality of improved emotional–behavioral regulation when aiming to improve EFs through different intervention programs.

Furthermore, as previous research has reported behavior regulation to be closely linked to social function (Kenworthy et al., 2009), and metacognitive competences of more importance for school performance (Carretti et al., 2014), our findings from both BRIEF and teacher interviews may indicate that the main advantage of such intervention programs will be related to children’s social function, rather than on academic outcome. However, improved social function may boost academic outcome, in the long run, as improved social competencies enhance cooperation needed for solving many of the tasks given in school settings. Egeland and Fallmyr (2010) have speculated that the BRIEF’s emotional regulation scale (a subscale of BRI) reflects the emotional and motivational aspects in EFs (i.e., hot EFs), in contrast to the less emotional items constituting the remaining scales in the BRIEF. In line with the interpretation by Egeland and Fallmyr (2010), improved BRI may reflect the necessities identified by Diamond (2014), that effective EFs training programs also help children to reduce stress, increase joy, make children feel they belong and improve physical fitness, i.e., in sum programs that not only will improve EFs and physical health, but also the children’s mental health (Diamond, 2014). This assumption coincides with the conclusions from a critical review of a similar creative arts/culture-based curricula interventions, highlighting the benefits for well-being, citizenship, work-related skills, and habits (Thomson et al., 2015). Thus, improved BRI in the intervention group may not only reflect less problems with behavioral regulation, but also, according to Egeland and Fallmyr (2010) improved emotional regulation skills, and better mental health.

### Metacognition

One potential interpretation of our results showing no greater improvement on MI in the intervention group, compared to the control group may be that potential improvements related to academic problem-solving activities (MI) may be more easily overlooked by teachers than the more overt behavior regulation competencies incorporated in the BRI. Previous research with clinical samples has shown that parents and teachers often report more behavioral symptoms, while children often report more symptoms about themselves than parents and teachers do regarding anxiety and depression (Faraone et al., 1995; Grills and Ollendick, 2002; Sciutto et al., 2004; Rothen et al., 2009; Skogli et al., 2013). Consequently, teacher ratings may be informative regarding behavioral regulation, but less sensitive regarding metacognitive competencies in children at school.

### Strengths and Limitations of the Study

Strengths of the current study is a global intervention specifically aimed at improving executive functioning and the use of an everyday EFs measure pre, post, and 6 months after intervention. Living in a society with low socio-economic differences and very high attendance to public schools, the study comprises a relatively representative group of pupils. The randomized controlled trial design and the implementation of both qualitative and quantitative data are also regarded as a strength. As the interventions were governed by teachers and artists in classrooms there is a possibility that interventions diverted slightly from how it was originally planned. The project manager visited each school to observe the sessions in order to check fidelity; however, no checklists or other means of checking fidelity were applied. A major limitation is using only teacher reports as outcome measure. This may cause some difficulties regarding a potential teacher–child disagreement. Due to this potential informant variance, it may be stated that what we actually measure is the teacher’s apprehension of the child and not the child’s capabilities. Therefore, improved EFs may more precisely reflect the teachers altered apprehension of the child. Teachers investment of time and energy to make the intervention work may also reflect how they rate their children after the intervention. However, as the effect was more visible after 6 months this is less likely. Other difficulties with the study are a relatively low *n*, and little control over confounding factors.

## Conclusion

In conclusion, the AoL program shows promising effects on behavioral self-regulation (BRI) improvement in children aged 6–9 years as reported both from teacher rating scales and interviews. The executive subfunctions underpinning social competencies rather than academic outcome seem to be most affected by the intervention. It remains to be seen if this in turn will improve academic functioning as well.

## Data Availability

The datasets generated for this study are available on request to the corresponding author.

## Ethics Statement

This study was carried out in accordance with the recommendations of the Norwegian Centre for Research Data with written informed consent from parents of all subjects. All parents gave written informed consent in accordance with the Declaration of Helsinki. The protocol was approved by the Norwegian Centre for Research Data.

## Author Contributions

PA collected the quantitative data material, did the statistical analyses, and made the first draft of the manuscript. MK did the interviews and the qualitative analyses. ES wrote parts of the manuscript. All authors contributed to the interpretation of both statistical and qualitative analyses, proofread and revised the manuscript, and gave approval to the publication.

## Conflict of Interest Statement

The authors declare that the research was conducted in the absence of any commercial or financial relationships that could be construed as a potential conflict of interest.
