# Supplementary material for: Art of Learning – An Art-Based Intervention Aimed at Improving Children’s Executive Functions
Source: Front Psychol. 2019 Jul 31;10:1769. doi: 10.3389/fpsyg.2019.01769 (PMC6685039; doi:10.3389/fpsyg.2019.01769)
Supplement: Supplementary file 1 [file Data_Sheet_1.PDF]

1 Warm Up

# Week 1 Session 1 – Dance Warm-Up

10'

**Objectives** To warm up different parts of the body following the verbal and visual instructions.

**Cognitive process** Inhibitory control, because learners have to be disciplined and resist distractions.

## instructions

1. **In the session today we are focussing on two things, movement and also getting to know each other. Before we start to do any physical activity, what should we do?** Give the learners time to respond. **Yes, we need to warm up so that our bodies are prepared for work. We are going to do our warm-up to music, and I will explain as we go along. Can everyone get into a big circle? Stretch your arms out; you shouldn't be able touch the people next to you in the circle.** Turn the music on. Perform all the moves as you are describing them.
2. **Now let's begin. Stretch your fingers, then move your hands back and forward, raise your arms, and back down again, then just one arm, twisting your hand around, then the same with your left arm. Now raise both hands above your head, bring your hands together, lower them and repeat. Keep your hands together and point them to the ceiling then bend over to your right, then bend over to your left.**
3. **Push both arms out, stretch your right leg back, stretch your arms out, then put them on the floor and stretch your left leg back, lower your body to the floor and look up.**
4. **Bring your head back down to the floor, then push up with your arms, come back down and kneel. Stretch out and sit back on your legs stretching your arms forward, then sit up and stretch both arms into the air putting your hands together and then lowering them. Sit back on your legs and then back up again.**
5. **Point your arms forward, then stretch them out as far as you can, then bring them back together but don't let them touch. Stretch them out again, then back together – but this time they touch. Repeat.**
6. **Crouch down and move your hands forward, then stretch your arms behind your body as far as you can and lift your legs up from the floor, and then stretch your arms forward in front of your body and your legs back as far as you can.** Turn the music off.

## reflection

- **Talk to a partner next to you. Share one thing that each of you noticed when you were doing the warm-up exercise.** Give learners a couple of minutes. The adults in the space should also reflect on what they observed or noticed and share their views, particularly in relation to learners staying on task, those that worked hard on concentrating and were not distracted, etc Get learners to share as many things as you can in the time left.

## scaffolding ideas

- You can modify the routine and also adapt it to the needs of the particular group. For learners with too much energy or those who are easily distracted, slow movements and focus on deep breathing can help them focus and settle. If learners lack energy, a faster, more energetic warm-up to upbeat music might be more beneficial.

## materials

- CD player or similar.
- Appropriate music – suitable copyright-free music provided.
- Video of the warm-up routine provided for the teacher or artist to watch beforehand to familiarise themselves with all the movements.

## set up

Open space (eg hall) large enough for everyone to sit or lie down.

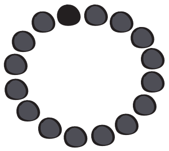

## tips

## 2 Main activity

# Week 1 Session 1 – Name Dance

40'

**Objectives**
To create a group movement piece by joining up individual pieces from the warm-up.

**Cognitive process**
Working memory, because learners have to use their short-term memory to recall their movements from the warm-up and incorporate them into this activity. This also involves cognitive flexibility, because they have to be imaginative about how they can join a number of small movements into a collaborative piece and switch their focus from working alone to working with others.

**Curriculum links**
Expressive arts: participation in performances and presentations. Literacy: Listening and talking and using information.

### instructions

1. **You will remember that at the start of the session I said that we were going to focus on two things. Can anyone remember what they were?** Give the learners time to respond. **Yes, we were going to focus on movement and on getting to know each other. So in this part of the session we are going to do both those things. I'd like to get us all to introduce ourselves and one by one we will go around the circle and say our names. When each person says their name, we should all repeat it. This is a call and response.** Do this clockwise. **That was easy!**
2. **We are going to do this again, but this time I want you to say your name and then perform a movement that you think characterises or represents you. This can be something you do often or enjoy, or how you feel; it's your choice. It can be just one simple movement. Not a still pose though – make it a movement. I'll say my name and show you my movement. And again we do the call and response, so you repeat my name and my movement.**
3. **So think for a minute about your own moves.** Give them thinking time. **Okay, are we ready? I'll start. My name is…** Say your name and perform a move, and remind them to repeat it after you. Then ask the learner next to you moving clockwise.
4. **Now I want you to form groups of three or four. In your groups, I want you to see how you can join your name movements together to make a collaborative or group movement piece. So it isn't about you doing your individual movements one after the other; you need to think about how you might connect them together. Each of you will perform the whole piece, and it needs to include all your moves, so the final piece will be something that represents all of you. First, share your moves again and think about how they might be connected. I will put some music on which will just be on repeat as that might help us.** Leave the groups to work things out for a while and look at how the groups are doing and identify those that are getting it so that they can share their work in progress with others.
5. **Now let's stop and see how we are getting on.** Get some groups to share. **What might we need to think of in creating our piece?** Prompts might include: the order of the moves, the position you start from and end, speed and tempo of the moves, varying the height of the moves (high, medium, low), the movement of different parts of our bodies, movements from small to large, where each of us stands. You might want to write these ideas down on a whiteboard or similar. **Remember that you all need to do all of the movement piece and not just the part that is about your name.**
6. **You'll have about twenty minutes to work on this and practice and we will share our movement pieces. Are there any questions?** Ensure everyone is on task and offer support where needed, or ask groups to look at how other groups are doing for inspiration. **So, time is up – let's share.**

### materials

- CD player with appropriate music, such as that provided for the warm-up activity.

### set up

Large open space (eg hall) for large group circle / small groups.

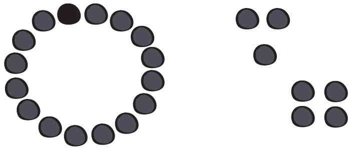

### tips

## 3 Reflection

# Week 1 Session 1 – Questions

10'

**Objectives**

To reflect on the strengths and areas of improvement of our group dance piece.

**Cognitive process**

Cognitive flexibility, because learners need to look at their work from a different perspective, analyse it and reflect on it in a concise way.

**instructions**

1. **Let's all sit in a circle – sit next to the people from your group. In your group, I want to you come up with one thing you thought was really good or strong about your movement piece and one thing you would do to improve it.** Give the groups a few minutes to reflect.
2. **Let's share first the good thing or strength.** Go around each group, reminding them to keep their answers brief.
3. **Next, share one thing you would do to improve your movement piece.** Go around each group, reminding them to keep their answers brief. Thank everyone for their hard work and their reflections.

### materials

→ None.

### set up

Large open space (eg hall) for large group circle.

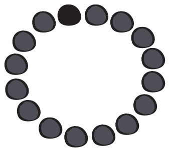

### tips
