# Supplementary material for: Art of Learning – An Art-Based Intervention Aimed at Improving Children’s Executive Functions
Source: Front Psychol. 2019 Jul 31;10:1769. doi: 10.3389/fpsyg.2019.01769 (PMC6685039; doi:10.3389/fpsyg.2019.01769)
Supplement: Supplementary file 2 [file Data_Sheet_2.PDF]

1 Warm Up

Week 2 Session 1 – Silent Walk

10'

Objectives To carefully observe familiar places in silence, looking for the best things about their school.

Cognitive process Inhibitory control, because learners have to resist speaking and getting distracted.

instructions

1. Ask learners to take a pen and paper and look around them. You want them to look at the familiar space in a new way and really think about it. They will have about 5 minutes to walk around the room slowly, and they should observe carefully and notice all the good things about this space, what they like about it, and why. They should think about all the different areas and objects, maybe notice things they have not noticed before, and write them down. However, they have to be completely silent throughout the short activity, observing through their sight and other senses and writing the observations down without talking. They can touch things as long as they are careful and return everything back where it was.
2. Ensure there is order and learners stay on task and take at least brief notes.

reflection

→ Let 2–3 learners share what they have written down, give arguments for why they think something is good, and reflect on how it felt to observe silently.

scaffolding ideas

→ Depending on learners' energy levels, you might wish to start with a very short physical activity that will energise them before moving on to silent observation.

materials

→ Paper, pen and clipboard for each learner.

set up

Large open space (eg hall) or the classroom to walk around freely.

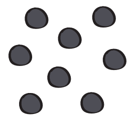

tips

2 Main activity

Week 2 Session 1 – School Advert 1

45'

Objectives

To look at the questions 'What is school for?' and 'What makes a school good?' from different perspectives in preparation for further work on analysing and presenting good things about their school.

Cognitive process

Cognitive flexibility, because learners have to think deeply from different perspectives. Emotional intelligence, because learners have to consider how different people feel about school and what their needs are.

Curriculum links

Health and wellbeing: mental and emotional wellbeing, expressing feelings and understanding others. Social wellbeing representing my community. Literacy: creating texts. Expressive arts: drama, creating, developing and sustaining a realistic character through voice, movement and language and develop ideas from a range of stimuli.

instructions

1. Learners need to form small groups of 4 or 5. Give each group the flipchart paper and marker pens. Ask them to have a little think about what school is for. They should talk to the other learners in their group and write down their ideas, with illustrations if needed. Give them 5 minutes, with a one-minute warning.
2. Let 2–3 learners share a couple of their ideas.
3. Now they should look at their ideas again and think about what makes a school good. Ask them to write their ideas down on paper. Give them 5 minutes, with a one-minute warning.
4. When everybody is finished, take pictures of all flipcharts. Ask them: if I posted their pictures on the school's website for all the teachers to see, do you think they would agree on everything, or maybe only some of these things? What about the head teacher? And your parents? Would they agree on your ideas about what makes a good school?
5. Ask the groups to act out a little conversation, with each of them adopting a role: a class teacher, a head teacher, a parent or a learner. Staying in character, they have to imagine they are being recorded for a TV talk show about schools, and they have to express their opinions on what schools are for and what makes a school good, from the perspective of that role. (If needed, discuss what a 'perspective' is.)
6. Encourage them to have a little think before they start and to really try to think as somebody else and imagine why their perspective and opinions could be a little different. Give them 5 minutes to divide roles and consider what their character might think – but because they will all have only very little time to talk as their slot on the TV show is so short, they need to prepare only a summary of the most important things they could say. Afterwards, they should start with the show going 'live'. They should all talk about what they (their character) thinks about the aims of schools and when a school is good. They can also compare their views, say they agree or disagree with somebody, politely, and react to what the others say. There is no TV show moderator, but they should imagine they are being filmed. You should walk from group to group and film pieces of their shows on a digital tablet. (continued on next page)

materials

- At least three sheets of flipchart paper for each group.
- 3–4 marker pens in different colours for each group.
- One tablet for the facilitator to take pictures and record video.

set up

Large open space (eg hall) suitable for working in small groups of 4–5 on the floor and in a large group circle.

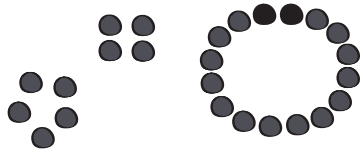

tips

instructions

7. (continued) Give them about 10 minutes for the activity. Move between groups, capturing pieces of the conversations. If it feels like learners are losing inspiration, ask them to swap roles and start a new TV talk show.

8. Explain that in order to get more people's opinions, they might find it interesting to interview their parents, before the next session. If they cannot talk to one of their parents/carers that day, it can be a grandparent, or an older sibling. They should imagine that they are interviewing for a school online magazine or a local newspaper, and the question they should ask is, 'What do you think makes a school good?' It can be a very short answer or a longer one. Encourage them to take notes of the most interesting things they hear so that they can remember them and share them at the beginning of the next session if they wish.

9. Tell them that all the work they have done so far, and what they learn from their relatives, will help them with some exciting work you will do together soon – shooting a video about their school and what is good about it.

variants

→ If you think children would work better sharing the same role within a group and preparing together their perspective and what they would say in the TV show activity, assign just one role per group, and they have to discuss this together and agree on the things they would like to say on the TV show.

scaffolding ideas

→ You could add a third question at the beginning for learners to reflect on: what helps us learn?

→ Instead of asking learners to pick one of the four roles for the role-play discussion (teacher, head teacher, parent, learner), you could ask them to draw blindly one of four pieces of paper with the respective roles written on them. You would have to prepare the set(s) ahead of the session.

→ If learners are struggling with ideas about what makes a good school, be ready to ask them questions to support their thinking. For example, if you were a head teacher, do you think it is important that staff work hard? From the point of learners, this could be, for example, questions about feeling safe, feeling respected by teachers, being able to learn a lot, being able to be yourself.

→ If you had more time for add-on activities, you could tell learners towards the end of the session that they will now get a chance to hear some opinions from other people too. Invite the head teacher or a senior staff member to come in and talk to learners briefly about what, in their opinion, school is for and what makes a good school. (If this is not possible, you could show a short video message shot beforehand.) If possible, have a short Q&A session with the head teacher and the teacher, to allow learners to ask questions relevant to the session topic.

tips

3 Reflection

Week 2 Session 1 – Yes / No Questions

5'

Objectives

To reflect on what they learnt in the session and the challenges of looking at schools from the point of view of adults.

Cognitive process

Cognitive flexibility, because learners have to reflect on the session and their challenges and express opinions considering different points of view.

instructions

1. Ask learners to go back into their small groups. They now have to try to reflect on the session, but they have to do this in a particular way. Taking it in turns, each member of their group has to come up with a question that they would like somebody from a different group to answer but that can only be answered with a Yes or a No response – so a closed question. The questions must relate to the session; for example, it could be "Do you really think school is about making friends" or "The teacher said school was about making us think about how we learn; do you think we do this enough at our school?". They should then ask this person, and the person should reply as honestly as they can.
2. Listen in to the work and offer support as needed.

materials

→ None.

set up

The same small groups.

tips
