# Supplementary material for: Art of Learning – An Art-Based Intervention Aimed at Improving Children’s Executive Functions
Source: Front Psychol. 2019 Jul 31;10:1769. doi: 10.3389/fpsyg.2019.01769 (PMC6685039; doi:10.3389/fpsyg.2019.01769)
Supplement: Supplementary file 3 [file Data_Sheet_3.PDF]

1 Warm Up

10'

Week 2 Session 3 – Speed Graffiti

Objectives

To recall relevant information from the previous day in a competitive and physical way.

Cognitive process

Working memory, because learners have to recall different words from the previous session. Cognitive flexibility, because they have to adapt quickly if their choice has been picked by someone else.

instructions

1. Remind learners of the activity from the previous day in which they had to come up with words characterising children and young people from Scotland. Ask them to form more-or-less equal queues in front of the flipchart papers on the wall.
2. The game will be something like a relay race. As a group, they have to remember as many words as they can from the previous session about what children and young people in Scotland are like. The person at the front in each group will have to run with their marker pen and write on their flipchart one word/expression, then run back and pass the marker pen to the next person. This person then needs to run and write a new word, and the game continues until 3 minutes are up.
3. Ask each group to count up the number of ideas they came up with. Congratulate the groups.
4. Ask the learners why you did this exercise (to warm up your bodies and your minds).

materials

- At least three sheets of flipchart paper, to be temporarily fixed to a wall with blu-tac, sellotape or similar.
- Marker pens in three different colours, one per colour to be given to each of the groups and spare ones to be placed underneath the flipcharts.
- Masking tape.

set up

Large open space (eg hall), mark out a start/finish line with masking tape, place flipchart paper on the wall and get learners into around five rows behind the line and facing the flipchart sheets on the wall.

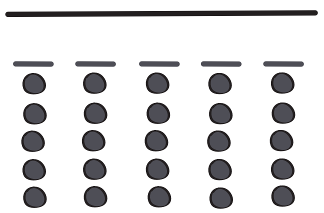

tips

2 Main activity

Week 2 Session 3 – What It's Like To Be… 2

35'

Objectives

To reflect on age-specific identity through a short drama piece, comparing positions and perspectives of children/young people and adults.

Cognitive process

Cognitive flexibility, because learners need to think about their lives and those of adults around them from different perspectives. Emotional intelligence, because learners explore other people's feelings, difficulties they are facing and what is important to them.

Curriculum links

Health and wellbeing: social wellbeing representing my community. Literacy: creating texts. Expressive arts: drama – creating, developing and sustaining a realistic character through voice, movement and language and developing ideas from a range of stimuli.

instructions

1. Get learners to think about words that in their opinion describe adults in their area. These can be the same or similar to those describing a child or a young person or also very different. They should think about their parents and other family members, their teachers and other people they know, what they are like, what they do very often, what their lives are like, what they enjoy, what problems they are facing, etc. Ask a few pupils to share their ideas and write the words on a whiteboard.
2. Encourage learners to think about the differences and similarities they think there are compared to children and young people. In what respects are adults different? Give different learners the chance to share their views.
3. Get learners into small groups, different to the ones in the previous session. In their groups, learners now have to prepare a very short drama piece of no more than 2 minutes about what it is like to be an adult in that area. Again, they can look at positives or negatives and take a serious approach or turn it into comedy (without being unkind or offensive). They only have 10 minutes to prepare this time though. Again, they can write down some ideas and note down what they agree on if they wish but do not have to.
4. Give learners a 3-minute warning and later a one-minute warning.
5. Every group performs their short piece in front of the rest of the class.

scaffolding ideas

- You could record each performance on a tablet/phone.
- If you had more time, you could include extra reflection. Write names of learners in each group on separate blank envelopes. After each performance, ask learners to write down on small pieces of paper what they liked about it. These pieces of paper go into the respective group's envelope. At the end of the session, when everyone has finished, give each envelope to the respective group for them to read the feedback they received.

materials

- Paper, pen and clipboard to write on if needed, one per group.
- A whiteboard or similar.

set up

Large open space for work in small groups of 4–6 learners each.

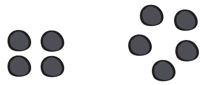

tips

3 Reflection

Week 2 Session 3 – Open-Minded Reflection

15'

Objectives

To reflect on the development of creativity skills.

Cognitive process

Cognitive flexibility, because learners need to reflect on the development of their skills in being open-minded.

instructions

1. Explain the use of the Open-Minded Reflection Tool. Ask learners to work in pairs but each should fill out their own reflection tool. They should think about which tick box best represents them and why, then include any evidence they have of being open-minded.
2. After 10 minutes come back together and ask learners to share how they found doing this and what they discovered. Ask them how we might develop our skills in being open-minded.

materials

→ Open-Minded Reflection Tool provided – one copy per learner.

set up

Pairs.

tips
