# Supplementary material for: Art of Learning – An Art-Based Intervention Aimed at Improving Children’s Executive Functions
Source: Front Psychol. 2019 Jul 31;10:1769. doi: 10.3389/fpsyg.2019.01769 (PMC6685039; doi:10.3389/fpsyg.2019.01769)
Supplement: Supplementary file 4 [file Data_Sheet_4.PDF]

1 Warm Up

Week 1 Session 2 – This is a...

15'

Objectives To come up with ideas of other things that a piece of cloth is like.

Cognitive process Cognitive flexibility, because learners have to use their imagination in thinking about familiar things in a new way.

instructions

1. **Let's all stand in a big circle. We are going to do another warm-up. Can anyone remember the reason why we did this yesterday?** Wait for responses. **To get our brains working. I have a piece of cloth and we have to think about different things that this cloth is like. When we compare things, or say that they are like something else – what is the word we use for this?** Wait for responses. **It is a simile.**
2. **So think for a moment about what this cloth is like – let your imagination run wild.** Give learners some thinking time. **So we are going to go around the circle and each one of us will say "This is a cloth and it's like..." We can also add some actions in, move the cloth, hold it in a certain way, give it a different shape, show what the thing would be doing. Do we all understand? Let's just have a go and I will start. "This is a cloth and it's like a manta ray gliding in the deep ocean."** Make the cloth glide like a manta ray. Pass the cloth to the person on your right and learners go one by one around the circle. Give reminders when learners don't start with "This is a cloth and it's like…" Go around the whole circle and thank learners for working hard.

refelction

- Give each learner a set of red, yellow and green cards. **Let's all sit down. You each have a set of cards, and in a minute we are all going to close our eyes and reflect. Imagine that we had to do this again. If you feel "Yes, I would be able to do that", what card would you choose?** Wait for a response. **You would pick up the green card. If you feel "I am getting there but I might need a bit of help", you would choose the yellow card. And if you feel "I would be stuck and need extra help", then you could choose the red card. Remember to be honest.**
- **So, let's all close our eyes and think of the card we would choose.** Give learners a minute to think. **Soon, I am going to count to three and tell you to open your eyes. When you open your eyes, you need to select the card you choose and then hold it high above your head. So now on the count of three, one two three, open your eyes, pick your card and show it above your head.** The cards will provide you with insights of which learners might need the most support with ideas during the exercise, and it may also be useful for putting learners into groups of mixed ability.

scaffolding ideas

- Learners could work in pairs or threes to come up with an idea rather than thinking on their own.
- If learners found it hard to come up with an idea, you could give them one or two clue cards, with pictures of what the cloth could be like, and come back to them after they have had some thinking time.

materials

- A medium-sized piece of plain cloth, preferably white/pale.
- Clue cards provided for the session.
- A set of red, yellow and green cards for each learner, provided.

set up

Large open space (eg hall) for large group circle.

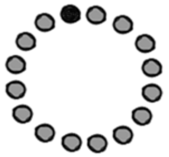

tips

2 Main activity

Week 1 Session 2 – Be Very Afraid

40'

Objectives

To create a verse for a poem on the theme of 'Be very afraid'.

Cognitive process

Cognitive flexibility, because learners have to be imaginative and inventive in thinking about familiar objects doing unfamiliar things.

Curriculum links

Literacy: tools for writing and creating texts.

instructions

1. **Today we are going to do some more poetry and I am going to read a poem called 'Be Very Afraid' by Carol Ann Duffy, a Scottish poet. I would like you to listen to the poem very carefully.** Read the poem out.
2. **What do you think the poem is about?** If there are no responses, tell the class the poem is about how your imagination can run wild, that ordinary things like toothpaste and pyjamas can come alive and do the most unreal or unimaginable things. **Like yesterday, we are going to create a poem today and work in groups to do that. We are going to imagine that when we leave home to come to school, things in the house come alive and do scary things. What objects in your house might come alive? Does the kettle steam a ghostly scream around the kitchen?**
3. **I have brought some objects in that you might find at home, and we are going to use them in our poems. I am going to give each of you an object and then put you into a group. When you go into your group, you have to think of your own first. Look at your object; imagine that it has come to life and that it does something scary, something that it wouldn't usually do.** Children are then put into groups of four (predetermined by the teacher). **Try to think on your own first, but if you are stuck, ask for help from your group.** Give the children 10 minutes to work on their ideas.
4. **Now we are going to develop our ideas. Each person in your group is going to use them to come up with two lines of a poem, and then together your group will have created a verse for a poem. The first line should be easy as it will always start with 'Be Very Afraid of the …'. So in my example, my first line would be something like 'Be very afraid of the kettle' and my second line would be 'which steams a ghostly scream around the kitchen'. I have a sheet for each of you so that you can write your two lines up. Any questions?** Give the children 10 minutes to work up their ideas. Remind them to help each other. Give them a 2-minute warning.
5. **So now each group is going to share their verse with the class. When the last person in each group has finished, we all say together 'BE AFRAID', as that is how Carol Ann Duffy ends each verse of her poem.** If time is running out, this might have to be done by dividing the class into two groups.

scaffolding ideas

- Ask learners to use their yellow and red cards to show they need help or support. If children are finding it difficult to come up with ideas, prompt them with the following: **What strange sound could your object make? What scary animal could your object be? Where could your object hide? How could your object frighten you?**

materials

- The poem 'Be Very Afraid' by Carol Ann Duffy, published, for example, in her book The Hat (this can be purchased here: www.faber.co.uk/9780571219667-the-hat.html).
- A wide range of small everyday objects (one for each learner); ideas include – key, CD, piece of pasta, peg, earphones, earing, cotton bud, string, paper clip, fork, sock, bus/train ticket, hair bobble, plaster, ice cube tray, sachet of sugar, straw, glove, ribbon, penny, bottle top, feather, post it note, tooth pick, ball, plastic toy, screw/nail, safety pin, elastic band, nail file, coaster, coat-hanger, button, candle.
- Print-outs (one per learner) of the 'Be very afraid' template, provided.

set up

Large open space (eg hall) for large group circle and groups of four to five.

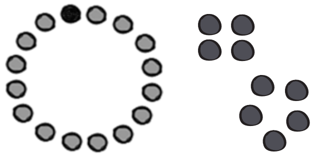

tips

3 Reflection

Week 1 Session 2 – Step In

5

Objectives: To reflect and take a step in when we agree with a statement.

Cognitive process: Cognitive flexibility, because learners have to reach a decision and then use their bodies to show their decision.

instructions

1. **Let's all come back together in a circle. In a minute I am going to say something, and if you agree with what I say, then you take a step forward into the circle. If you don't agree, just stay where you are.**

   I like watching television.

   Making poems is difficult.

   I know where Carol Ann Duffy was born.

   I tried hard with my ideas.

   To support further reflection, you might want to count how many learners step forward for each of the reflection statements.

2. Congratulate everyone for their hard work and find a suitable place to display the verses created by each group.

materials

- None required.

set up

Large open space (eg hall) for large group circle.

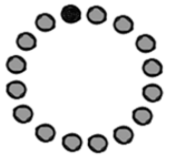

tips
