# Supplementary material for: Art of Learning – An Art-Based Intervention Aimed at Improving Children’s Executive Functions
Source: Front Psychol. 2019 Jul 31;10:1769. doi: 10.3389/fpsyg.2019.01769 (PMC6685039; doi:10.3389/fpsyg.2019.01769)
Supplement: Supplementary file 5 [file Data_Sheet_5.PDF]

1 Warm Up

Week 1 Session 2 – Don’t Clap This One Back

10’

Objectives

To recognise different rhythms and remember instructions associated with some of them when clapping.

Cognitive process

Inhibitory control, because pupils have to clap rhythms as shown and in time and think before acting, recognising the rhythm that cannot be clapped back. Working memory, because pupils have to remember new rhythms, the differences between them and recognise the instruction that accompanies some of them.

instructions

1. Right, let’s get warmed-up and ready! Let’s all start by simply walking to the rhythm of four beats. I’ll count one to four, and all you have to do is walk one step per beat, starting with your right foot, so ‘one, two, three, four’ would be ‘right, left, right, left’. You can walk in any direction as long as you don’t bump into each other. But make sure you listen to me and keep following the beat. **Start counting slowly, increasing speed, then go back from very fast to normal and finally very slow counting, taking no more than a minute or two.**
2. Good, now let’s all sit in a big circle. I’ll clap some rhythms and all you have to do is repeat after me. **Do simple four-beat rhythms, increasing difficulty. Some examples could be:**
3. Now I’ll teach you one rhythm that I want you to remember, and to help us remember it, it comes with the words ‘Don’t clap this one back’. And every time you hear this rhythm, you can’t clap it back. Clap the following rhythm, accompanying it with the corresponding words:

1	2	3	4
Don’t	clap	this one	back
♩	♩	♫	♩

4. **Get pupils to practice the clap through call and response a few times.** Good, and now we’ll do different rhythms again, and you have to repeat them after me, all but the one we learnt to the words ‘Don’t clap this one back’. When you hear that one – I’ll do it without words this time – you have to remember not to clap, just wait for four beats until I give you a different rhythm. Is that all clear?
5. **Clap a mixture of different rhythms including the one above, keeping the activity fluid, without breaks.**

materials

→ None.

set up

Large open space (eg hall) for large group circle.

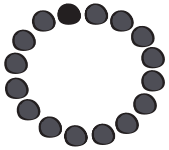

tips

1 Warm Up

Week 1 Session 2 – Don't Clap This One Back

10'

reflection

- **So we are going to do a quick reflection on that warm-up but we are going to do this using our voices and high and low notes. Can anyone share a high note?** Give pupils the opportunity to respond and if no one does, share a high note, and then share a very high note, if someone really really enjoyed it. **Can anyone share an example of a low note?** Again give pupils the opportunity to respond first.
- **Can we use our voice to go from a high note and then all the way down to a low note and then back up again? Try that.**
- **So now I want you to think about whether you enjoyed the warm up. A high note means you enjoyed the warm-up and a low note means you didn't enjoy the warm-up. Let's try and hold that note but let's also listen to the sound we make as a class. Ready?** Listen to the sound the class makes. **So, hands up if you think more of the class enjoyed the warm-up than didn't.** If many pupils did not enjoy the activity, address this.
- **Let's try another. This time I want you to think about whether you feel in the warm-up you got better at repeating the rhythms and knowing when to clap and when not to clap. The higher the note, the more improvement you think you made. What might my note be like if I feel I improved a lot?** Give pupils the opportunity to respond first. **If you feel you stayed about the same, which is also fine, your note would be somewhere in the middle, but go higher depending on how much you feel you have improved. Let's try and hold our tone again while listening to the sound we make as a class too.** Listen to the sound the class makes. **Hands up if you think most of the class improved.**

scaffolding ideas

- If the pupils found the activity easy, one or two extra rhythms with specific instructions could be introduced:

1	2	3	4
Put your	hands	on your	head
♫	♩	♫	♩
Stand	up and	turn a-	round
♩	♫	♫	♩

You could also ask the group to come up with their own rhythms with instructions.

tips

2 Main activity

Week 1 Session 2 – Louisiana Mud Slap

40'

Objectives

To perform a group rhythm routine, with two pairs performing different moves at different times.

Cognitive process

Inhibitory control, because pupils have to pay close attention, focus and follow the instructions exactly to match movements in time with their partners. Working memory, because pupils have to be able to recall the pattern of moves in the correct order.

Curriculum links

Expressive Arts: Contribute to a performance.

instructions

1. Ahead of the session, watch the video provided to understand what the routine should look like.
2. **Right, everyone, let's stay in a circle and I'll teach you a rhythm routine.** Perform this slowly and comment, then teach it adding one beat at a time until they are all confident.

1	2	3	4	5	6	7	8
Stamp Stamp	Slap Slap	Clap	Right Hand	Clap	Both hands	Clap	Left Hand

3. **This isn't a challenge to see how quickly you can do it, it's about keeping the beat steady. Remember there are two stamps and two claps in the first two beats, but only one action per beat after that.**
4. **Okay, that looks great, now let's do it in pairs. The right hand and left hand movements now become a 'high five'. Remember to keep the beat steady and you can count out loud to help.**
5. **Join two pairs together to make a group of four. Keeping the same partners, the two pairs stand side by side and do it at the same time so that they are listening to each other and working together to keep the beat steady.**
6. **Ok now we're going to add a little twist. Keeping the same partner and facing each other, we're going to arrange ourselves in a square shape so that each person is one corner of the square.** Demonstrate this with one group.

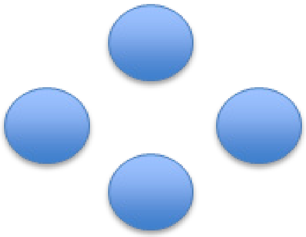

materials

- The videos provided, to be watched ahead of the session.

set up

tips

instructions

7. We're going to do the mud slap in our group of four, but if we're all doing it at the same time there could be a problem, what might happen? **(Pupils' hands will get tangled up when doing the hand claps with their partner).**
8. Ok, your challenge is to figure out how to get around that! **(There could be different solutions, eg, one pair could do a low high five, they might clap hands with a new person etc.)**
9. Remember to keep the beat steady!

tips

scaffolding ideas

- ➔ Staying in the square shape, pair A starts the routine from the first beat (A1) and on the fifth beat (B1) pair B begins the routine, similar to two groups singing a song (like London's Burning) as a round. This means that when pair A is doing the clap on the fifth beat (B1), pair B will be doing two stamps as the first beat (A1) of their routine.
- ➔ As they continue to repeat the routine, they will find that on beats 4 and 8 they will now meet the hand(s) of the person next to them and not opposite.

A1	2	3	4	B1	2	3	4
Stamp Stamp	Slap Slap	Clap	Right Hand	Clap	Both hands	Clap	Left Hand

- ➔ When Pair A get to beat 4 'right hand', Pair B will be at beat 8 'left hand', meaning that pair A's right hands will meet the left hands of Pair B.
- ➔ Similarly, when Pair A get to beat 8 'left hand', Pair B will be at beat 4 'right hand', meaning that pair A's left hands will meet the right hands of Pair B.

3 Reflection

Week 1 Session 2 – Reflection

10'

Objectives

To reflect on the group's work using notes of different heights.

Cognitive process

Cognitive flexibility, because pupils need to reflect on their work in non-binary way and negotiate this as a group.

instructions

1. **So, working in your groups, we are going to do a reflection like we did after the warm-up. Can anyone remember what we did?** Give pupils the opportunity to respond before reminding them.
2. **I'm going to ask you to think about your work as a group in the previous activity, Louisiana Mud Slap. I will make a statement and then in your group you have to decide how high or low your notes will be – the more you agree with what I say the higher your notes. You need to think about my statement, have a short discussion and then practice so that you are all using the same notes. So let's start.** You might want to record the next parts for reflection later.
3. **First, 'doing this session was REALLY EASY' – and remember, the more you agree with this, the higher your group note will be.** Give pupils time and then get each group to share their notes and also ask some groups to share the reasons why they chose their note.
4. **Second, 'we made lots of MISTAKES' – the more mistakes you made, the higher your group note will be.** Repeat the sharing process but remind pupils that making mistakes is not a bad think, it is an important part of learning and allows us to continue to improve.
5. **And last, 'we worked HARD as a group' – the harder you worked, the higher your group note will be.** Repeat the sharing process and stress the importance of hard work and effort in learning, in trying different strategies and taking steps to improve.

materials

→ None.

set up

Groups of 5 to 8 from the previous activity.

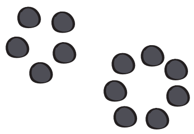

tips
