# Supplementary material for: Art of Learning – An Art-Based Intervention Aimed at Improving Children’s Executive Functions
Source: Front Psychol. 2019 Jul 31;10:1769. doi: 10.3389/fpsyg.2019.01769 (PMC6685039; doi:10.3389/fpsyg.2019.01769)
Supplement: Supplementary file 6 [file Data_Sheet_6.PDF]

1 Warm Up

Week 2 Session 3 – Changing Spaces

10'

Objectives To notice change, both deliberate and random.

Cognitive process Inhibitory control, because learners need to stay focused and resist distractions. Working memory, because learners need to compare their previous knowledge and observations with new ones to spot differences.

instructions

1. Start in the indoor space. Ask learners to look around for two minutes, notice interesting details and try to remember exactly what the space looks like.
2. Lead learners to the outdoor space. Ask them what they like about the indoor space. Then ask them to look around and say what they like about this outdoor space. This should only take 2–3 minutes. In the meantime, one adult needs to stay in the indoor space and change four things – this can be closing a window, moving a desk, rearranging pictures on the wall, etc.
3. Return to the indoor space and ask learners to work in pairs and spot the four changes.
4. Return to the outdoor space and ask learners to spot any changes that happened naturally, without the other facilitator making them. If they are struggling to identify enough, give them hints and tips – this could be the different position of clouds, birds, shadows, wind bending trees, passers-by or cars moving, doors and windows getting open/shut, etc.
5. You can conclude by saying that the world and life is full of changes big and small, and we make some of these ourselves, some are done deliberately by other people, and many are happening around us all the time.

materials

→ None required.

set up

Two different spaces not far from each other, one outdoors or with views of the outdoors and one indoors in which things can be changed quickly (e.g. objects moved around).

tips

2 Main activity

Week 2 Session 3 – Portrait of Change

45'

Objectives
To reflect on things that change us and express them visually.

Cognitive process
Cognitive flexibility, because learners need to look at things and their lives from new points of view and keep adapting to new triggers in the game. Emotional intelligence, because learners reflect on how different people and situations make them feel and on feelings of the people portrayed in the video.

Curriculum links
Health and wellbeing: self-awareness, self-worth, meeting challenges, managing change, spiritual and mental wellbeing. Art and design: recording and representing experiences.

instructions

1. Give each learner the flipchart with the face outline. Explain that in this activity, you will give them the opportunity to think hard about change. They will all be drawing their own 'Portrait of Change'. They will think about and see and hear different things and will draw how it affected them or made them feel. They do not have to insert eyes, nose or mouth – these can be inserted later to represent a particular change or emotion, but it is not necessary. They can draw both inside the face and around it; it does not have to look like a realistic face when it is finished. They should not focus on getting each drawing done perfectly – it is more important to draw the right things, what they think and feel.
2. First, start by exploring what learners think changes them – how they think, feel or look – all the time, every day or very often. This could be physical changes or also one's opinions, emotions, personality, habits, etc.; the changes can be permanent or temporary, immediate or observable later, long- or short-term. Let learners share their ideas, or help them with some examples (e.g. food, exercise, books, friends, fashion accessories, hobbies you practice regularly…). These should not be drawn yet; first lead a short open conversation about change and types of change.
3. Now ask them to take their template and draw some of the things that they think changed them in a positive way in the past few years, since they started school. Tell them that they only have about two minutes for the drawing.
4. Next, ask them to draw the people that changed them the most in the past few years. Again, give them about two minutes.
5. They should now draw positive experiences that changed them, something good that happened to them or they did.
6. Show the video provided (of a man with no legs dancing with a ballerina). Ask them to share how it made them feel, how it affected them. If it made them sad, why? How is the man in the video feeling, what emotions does he show? What about the ballerina?
7. Learners draw how watching this video changed them, what it made them feel. Emphasise that there is no right or wrong answer. (continued on next page)

materials

- Flipchart templates with an outline of a face with shoulders, one per pupil.
- Enough markers and felt pens of different colours to be distributed around the room and shared.
- Short video available here: https://www.youtube.com/watch?v=QgUT0Ufmkbk.
- Laptop and projector or similar to show the video to the whole class.
- Music provided and music player to play the song to the whole class.

set up

Large open space (eg hall) with enough space for everyone to draw on a sheet of flipchart paper each.

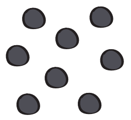

tips

instructions

8. (continued) Play the music provided and ask learners to draw how that made them feel and changed them. Again, no right or wrong answer.
9. Now, ask learners to think of difficult experiences they had in the past few years that affected them strongly and draw one or two. These will be the last additions to their portrait.
10. Thank learmers for their hard work. They have created their 'portrait of change', showing their journey in the past few years, what affected them a long time ago or also earlier in the session. This is a glimpse of who they are at this specific moment, what made them the person they are today, and now they are sharing it with whoever looks at the picture.
11. Learners now in pairs swap their completed portraits and ask questions about them. They should not merely comment on what they like or how something is drawn but ask for more information or perhaps give suggestions through questions.

scaffolding ideas

→ If unable to play the video provided, find another video/audio recording or picture that would enable learners to explore emotions and emotional responses – you could look at topics such as equality, disability, 'otherness'/'them and us', life joy, life purpose, abilities and gifts, pity, guilt, etc., or other topics that are relevant generally or for that specific group and time and that could lead to a deep discussion.

tips

3 Reflection

Week 2 Session 3 – Scale Game

5'

Objectives

To reflect on the topic of change, wrapping up learning from earlier in the session.

Cognitive process

Emotional intelligence, because learners discuss their emotional responses to different types of change in their lives.

instructions

1. Start by talking about how everything is changing you all the time, and sometimes the change is smaller, sometimes larger; some changes can be seen by other people easily and some cannot.
2. Ask learners to answer the question 'Is change good or bad?' by finding their place on an imaginary scale/line, with one end of the line being 'good' and the other 'bad' and the middle 'neither good nor bad, or both'. They can stand on either end or anywhere between to express their view.
3. Ask a few learners to explain why they are where they are on the scale.
4. Discuss with the whole group:

- When might change be a good thing? Can you think of examples of change when it was a good thing?
- When might change be a bad thing?

materials

→ None required.

set up

Large open space (eg hall) for learners to find a spot on a long line from wall to wall, and a large group circle.

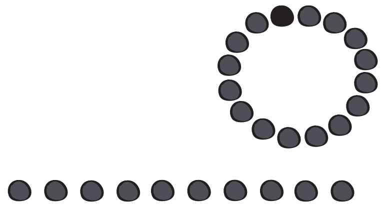

tips
