# Supplementary material for: Art of Learning – An Art-Based Intervention Aimed at Improving Children’s Executive Functions
Source: Front Psychol. 2019 Jul 31;10:1769. doi: 10.3389/fpsyg.2019.01769 (PMC6685039; doi:10.3389/fpsyg.2019.01769)
Supplement: Supplementary file 7 [file Data_Sheet_7.PDF]

Appendix 1. Art of Learning – professional learning programme

Day 1

Time	Session	Led by:	Notes:
**08.45 – 09.15**	**Set up**		Room set up
**09.15 – 09.30**	**Coffees to be available on arrival**		Welcome delegates
**09.30 – 09.35**	**Welcome and overview of the day**		
**09.35 – 10.05**	**Overview of AoL**		
**10.05 – 10.45**	**Overview of participating schools**		
**10.45 – 11.00**	**Coffee**		
**11.00 – 11.15**	**Exercise: Free Writing**		**FREE WRITING – GETTING TO KNOW YOUR SCHOOL**<br>Useful for: Reflecting and benchmarking; idea generation.
**11.15 – 11.45**	**Exercise: The River**		**THE RIVER**<br>We use the outcomes from the Free Writing exercise to create the content for The River. The River creates possibilities for personal ideas and reflections to feed into a wider group discussion.<br>Useful for: Reaching agreement, beginning to develop a shared language or understanding, giving up ideas and sharing control. It helps to build a picture of where the group is at and what is important.
**11.45 – 12.30**	**Presentation: Why are School Leaders Important?**		Why school leaders are important – what the evidence says (best evidence synthesis). The Role of the artists in acting as a critical friend /mediator in the school and with its leadership.
**12.30 – 13.15**	**Lunch**		
**13.15 – 13.30**	**Overview of the Art of**		

	**Learning Implementation Plan**		
**13.30 – 14.30**	**Art of Learning Evaluation Tools**		
**14.30 – 15.00**	**Exercise: Roles and Scenarios**		**ROLES AND SCENARIOS**<br><u>Useful for:</u> Allowing participants to consider some of the possible challenges and dilemmas within the Art of Learning and the role that they might play in finding solutions or in supporting other partners in the process to do the same, deepening the understanding and embedding the roles and responsibilities of the partners within the programme.<br>This activity is designed to facilitate discussions in groups of three. These groups will come together during the reflection to share their ideas and suggestions as to what the artist should do.
**15.00 – 15.15**	**Coffee**		
**15.15 – 16.30**	**Exercise: Hanging out the Dream Plan**		**HANGING OUT THE DREAM PLAN**<br><u>Useful for</u>: how artists will interface with the schools, preparing schools for artists to arrive, supporting the teachers on an ongoing basis - how will they connect with the participating teachers and school leaders preparing schools for the artists arrival
**16.30**	**End**		

Day 2

Time	Session	Led by:	Notes:
**08.30 – 09.15**	**Set up**		Room set up
**09.15 – 09.30**	**Coffees to be available on arrival**		Welcome delegates and give them their folders, name badges
**09.30 – 09.35**	**Welcome and introductions**		Brief welcome
**09.35 – 09.45**	**Warm up exercise**		Keepy uppy
**09.45 – 10.45**	**Presentation: Executive Functions**		What are EF's how do they relate to creativity skills and why are they important?<br>Include reference to links to EF's – mapping across
**10.45 – 11.00**	**Coffee**		
**11.00 – 12.30**	**Exercise: Session Plans**		**EXPLORING THE ART OF LEARNING CONTENT**<br>Useful for: Exploring in more details the content for each artform and how we will interact with it.
**12.30 – 13.15**	**Lunch**		
**13.15 – 14.30**	**Exercise: Collaborative Planning**		**COLLABORATIVE PLANNING**<br>Resources needed:<br>• AoL session plans<br>• Blank session plan – warm up, main activity and reflection<br>• Curriculum area<br>• Location in the school<br>• Executive function/creativity skills<br>• Fairy tale
**14.30 – 14.45**	**Presentation: Mental Models**		Share mental models information sheet and PPT
**14.45 – 16.00**	**Exercise: Role on the Wall**		**ROLE ON THE WALL**<br>Useful for: Exploring the connection between narrative and feelings to explore handing over and embedding practice

**16.00 – 16.30**	**Open Discussion**		Open discussion on the role of the artist, agreement on practical arrangements and next steps
**16.30**	**End**		
